# The impact of decellularization methods on extracellular matrix derived hydrogels

**DOI:** 10.1038/s41598-019-49575-2

**Published:** 2019-10-17

**Authors:** Julia Fernández-Pérez, Mark Ahearne

**Affiliations:** 1 Department of Mechanical and Manufacturing Engineering, School of Engineering, Trinity College Dublin, the University of Dublin, Dublin, Ireland; 2 Trinity Centre for Biomedical Engineering, Trinity Biomedical Science Institute, Trinity College Dublin, the University of Dublin, Dublin, Ireland

**Keywords:** Biomedical engineering, Biomaterials, Biomaterials, Bioinspired materials

## Abstract

Tissue-derived decellularized biomaterials are ideal for tissue engineering applications as they mimic the biochemical composition of the native tissue. These materials can be used as hydrogels for cell encapsulation and delivery. The decellularization process can alter the composition of the extracellular matrix (ECM) and thus influence the hydrogels characteristics. The aim of this study was to examine the impact of decellularization protocols in ECM-derived hydrogels obtained from porcine corneas. Porcine corneas were isolated and decellularized with SDS, Triton X-100 or by freeze-thaw cycles. All decellularization methods decreased DNA significantly when measured by PicoGreen and visually assessed by the absence of cell nuclei. Collagen and other ECM components were highly retained, as quantified by hydroxyproline content and sGAG, by histological analysis and by SDS-PAGE. Hydrogels obtained by freeze-thaw decellularization were the most transparent. The method of decellularization impacted gelation kinetics assessed by turbidimetric analysis. All hydrogels showed a fibrillary and porous structure determined by cryoSEM. Human corneal stromal cells were embedded in the hydrogels to assess cytotoxicity. SDS decellularization rendered cytotoxic hydrogels, while the other decellularization methods produced highly cytocompatible hydrogels. Freeze-thaw decellularization produced hydrogels with the overall best properties.

## Introduction

The extracellular matrix (ECM) is primarily composed of structural and regulatory proteins and polysaccharides and is generated and maintained by cells. Many cellular functions, such as proliferation, migration or differentiation are regulated by the ECM^1^. Each organ and tissue is composed of a distinctive ECM, in its biochemical composition and structural organization. The properties of ECM are important in the fields of tissue engineering and regenerative medicine, which often aim to replicate the composition and structure of the ECM. By using synthetic or natural materials, three-dimensional scaffolds can be fabricated to repair or restore damaged organs and tissues.

One popular approach to generating scaffolds that try to imitate the tissues or organs ECM characteristics is to use decellularization. This technique involves the removal of cellular components from a tissue so that only the ECM remains. Many methods have been examined for performing decellularization and these can be divided into three main categories: physical, chemical and biological^2^. Physical methods include freeze-thawing cycles^3–6^, high hydrostatic pressure^7–9^ or supercritical CO_2_^10–12^. Chemical agents can involve ionic detergents, such as sodium dodecyl sulphate (SDS)^13,14^ or sodium deoxycholate^15^; non-ionic detergents, such as Triton X-100^16^; hypertonic or hypotonic salt solutions, such as sodium chloride^17,18^; and acids and bases, such as peracetic acid^19^ or ammonium hydroxide^20^. Enzymes such as trypsin, dispase and phospholipase A2 have been used as biological methods for decellularization^21,22^. Furthermore, nucleases, such as DNAse, are used to promote the fragmentation of residual DNA into <200 bp fragments in order to minimize immunological responses^2^. Extensive research has been completed to optimize these decellularization procedures to allow for maximal cell removal and minimal ECM damage for each tissue/organ.

One difficulty associated with some decellularized tissues is their limited potential for recellularization. For many tissue-engineering applications, healthy cells need to be embedded into the ECM to generate a functional and viable tissue. To overcome this problem decellularized organs and tissues can be transformed into hydrogels that allow cells to be encapsulated throughout their structure. These hydrogels can then be used as injectables for minimally invasive delivery into irregular spaces^23–28^ and for 3D printing of scaffolds^29–34^. Since the first report of ECM-derived hydrogels in 1998^35^, over 70 papers have appeared in the literature describing the use of ECM-derived hydrogels from a wide variety of organs^36^. ECM-derived hydrogels have been under investigation to treat several medical conditions. These include type 1 diabetes, where the hydrogel provided a matrix to delivery cells to the pancreas^27^; myocardial infarction by replacing damaged cardiac tissue^23^, skin wounds^37^, and keratoconus by using the ECM to 3D bioprint a corneal stromal substitute^38^. Despite the increasing interest in such hydrogels, the effect of different decellularization methods on the final hydrogel characteristics has not been widely studied.

The aim of this study was to examine the impact of three different decellularization protocols on ECM-derived hydrogels obtained from porcine corneas. Two detergent-based techniques (SDS and Triton X-100) and a freeze-thaw cycling technique were used to decellularize corneas and hydrogels were fabricated from the resulting ECM. The impact of these decellularization protocols were evaluated in terms of biochemical composition, transparency, gelation kinetics, mechanical properties and cytocompatibility.

## Results

### Biochemical characterization of decellularized material

**Figure 1:**
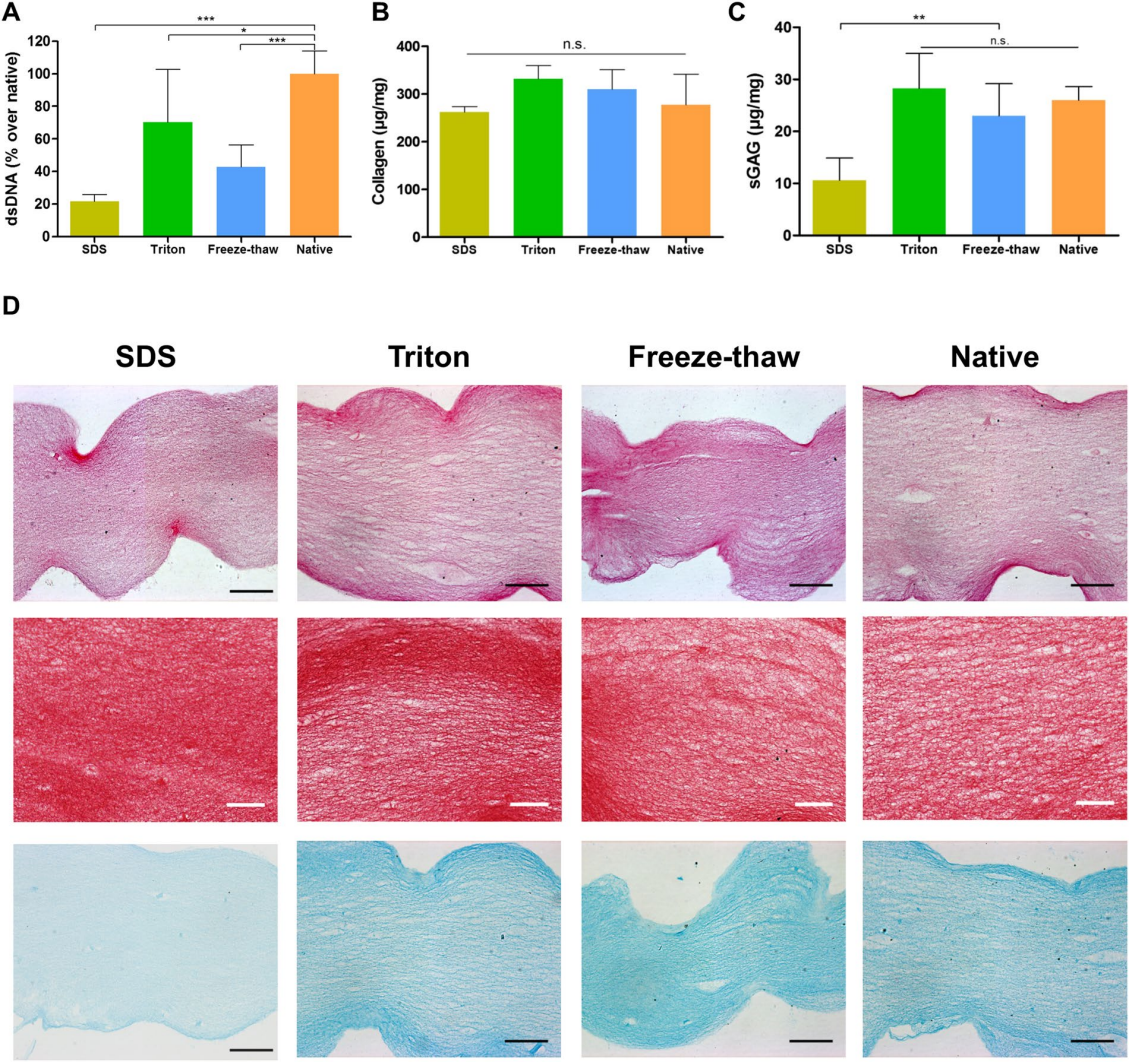
Evaluation of decellularization of ECM-derived hydrogels: (**A**) Quantification of dsDNA, (**B**) Collagen, and (**C**) sGAG; *p < 0.05, **p < 0.01, ***p < 0.001; (**D**) histological examination of hydrogels, stained with haematoxylin and eosin, picro-sirius red and Alcian blue; black scale bar = 100 µm, white scale bar = 50 µm.

The biochemical composition of the fabricated ECM hydrogels was analysed. PicoGreen was used to quantify DNA remnants, collagen content was measured by hydroxyproline quantification and sulphated glycosaminoglycans (sGAG) were quantified by dimethylmethylene blue assay (DMMB). All decellularization methods led to a significant reduction in DNA when compared to the non-decellularized control, i.e. hydrogels from native corneas (Fig. 1A). Collagen levels remained constant in all treatments (Fig. 1B). sGAG levels were maintained when decellularization was performed with Triton or the freeze-thaw methods, while SDS resulted in significant loss of sGAG (Fig. 1C). Furthermore, histological examination appeared to validate these results (Fig. 1D). Staining with haematoxylin & eosin confirmed the absence of cell nuclei after decellularization. Dense collagen was observed after picro-sirius red staining across all samples. Alcian blue staining showed the presence of sGAG in all hydrogels with a noticeable reduction in staining for the SDS treated group.

**Figure 2:**
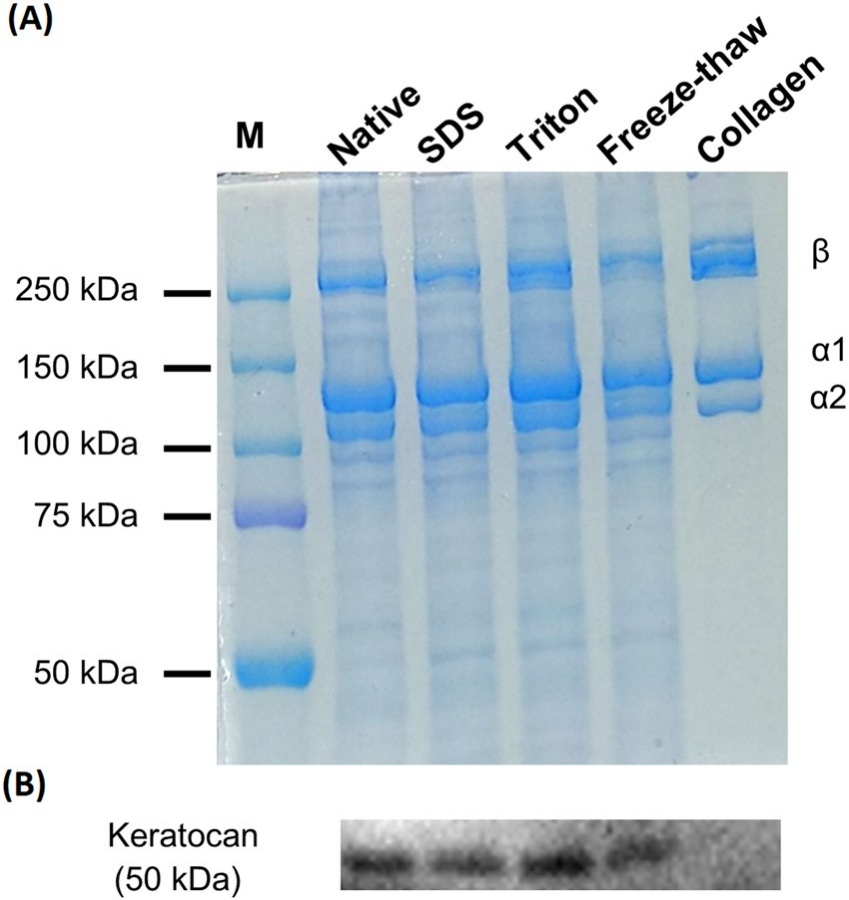
(**A**) Biochemical composition of ECM-derived hydrogels via SDS-PAGE (7%); (**B**) western blot against keratocan; M = molecular weight ladder.

Further analysis of the composition of ECM-derived hydrogels was performed using SDS-PAGE and western blotting. SDS-PAGE showed the presence of collagen chains β, α1 and α2 for all conditions. Gamma chains were too heavy to be detected in a 7% polyacrylamide gel. Other lighter proteins were detected in the ECM-derived material lanes but not in a pure collagen type I control isolated from rat tail (Fig. 2). Immunodetection via western blot detected the presence of the corneal proteoglycan keratocan in all ECM-derived materials, independent of decellularization method, but not in rat tail derived collagen.

### Light transmittance

**Figure 3:**
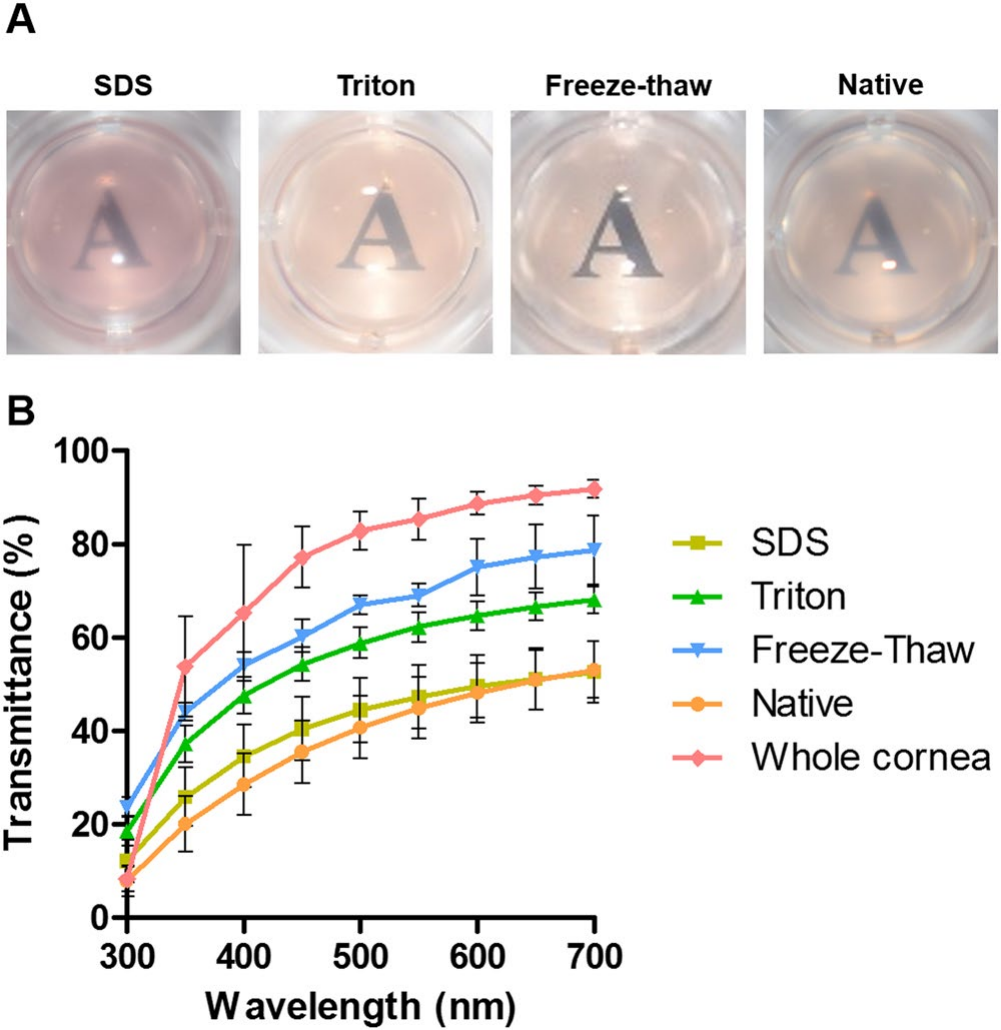
Transparency analysis of ECM-derived hydrogels: (**A**) Macroscopic appearance with hydrogels placed over printed text; (**B**) light transmittance quantification over the visible spectrum of light.

Since ECM from cornea was used to fabricate the hydrogels in this study, it is necessary to examine the transparency of the hydrogels since this is required for corneal tissue engineering. Transparency was measured by quantifying the light absorbed by the material and from this calculating the amount of light transmitted through each sample. As seen in Fig. 3, all hydrogels allowed light to pass through them, although hydrogels decellularized using SDS and the native hydrogels were cloudier in appearance. Quantitatively, all hydrogels presented at least 50% light transmittance at the end of the visible spectrum. Hydrogels decellularized using the freeze-thaw method were the most transparent with transmittance values above 70%. These values are only slightly lower than full thickness porcine corneas.

### Gelation kinetics

**Figure 4:**
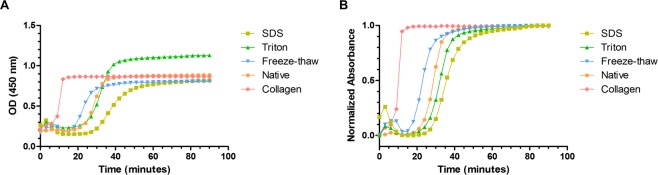
Gelation kinetics of ECM-derived hydrogels via turbidimetric analysis: (**A**) Raw values; (**B**) normalized data.

Gelation kinetics of ECM hydrogels were analysed by turbidimetric analysis. This technique is based on the increased in turbidity, and thus absorbance, experienced during collagen self-assembly. All samples presented a sigmoidal profile and gelled after a lag period or t_lag_ (Fig. 4). All treatments yielded hydrogels which started gelling after a longer lag phase than pure rat tail collagen (t_lag_ 7.93 ± 0.55 minutes). Freeze-thawing produced the earliest gelling material of all treatments (t_lag_ 16.43 ± 0.37 minutes), while SDS treated hydrogels took the longest to gel (t_lag_ 27.53 ± 1.36 minutes). However, there was no statistically significant difference between the different speeds at which the ECM-derived materials gelled. All values are displayed in Table 1.

**Table 1:** Turbidimetric analysis results of ECM-derived hydrogels. Average ± SD.

Condition	*S*	*t*_*1/2*_ *(min)*	*t*_*lag*_ *(min)*
SDS	0.061 (±0.006)	35.75 (±1.86)	27.53 (±1.36)
Triton	0.070 (±0.003)	32.14 (±0.33)	24.94 (±0.28)
Freeze-thaw	0.075 (±0.002)	23.11 (±0.35)	16.43 (±0.37)
Native	0.090 (±0.002)	28.04 (±1.51)	22.51 (±1.37)
Collagen	0.235 (±0.026)	10.08 (±0.33)	7.93 (±0.55)

### Rheology of ECM hydrogels

**Figure 5:**
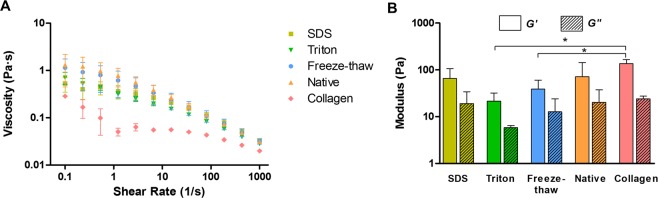
Rheology analysis of ECM-derived hydrogels: (**A**) Viscosity measurements at increasing shear rates; (**B**) storage modulus (G′) and loss modulus (G′); *p < 0.05.

Rheology was utilized to assess mechanical characteristics of the hydrogels. Increasing shear rates were used to calculate the viscosity at 15 °C, quite below gelling temperature. Shear thinning properties were observed in all pre-gel solutions, regardless of decellularization treatment (Fig. 5A). Storage modulus (*G*′) and loss modulus (*G*′) were determined by following the gelation kinetics at 37 °C over time at a fixed frequency of 1 rad/s and 5% strain. All hydrogels had similar moduli values with no statistical significance among decellularization treatments (Fig. 5B). Only the Triton and the freeze-thaw groups were significantly weaker than the rat tail collagen hydrogels.

### Evaluation of hydrogel ultrastructure

**Figure 6:**
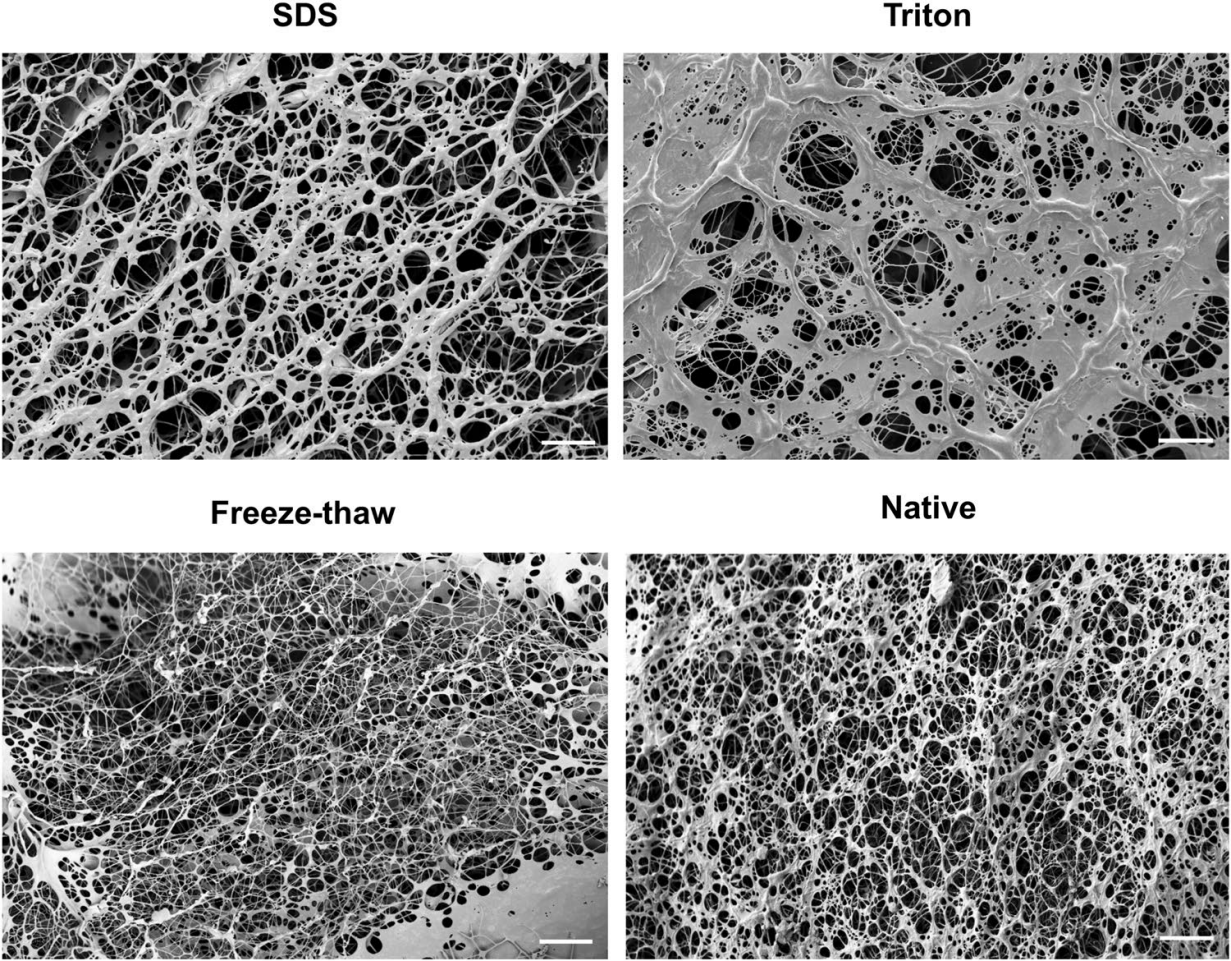
CryoSEM micrographs of ECM-derived hydrogels at ~1000x; scale bar = 10 µm.

CryoSEM was employed to study the structure of the hydrogels in the least disruptive way. Samples were snap frozen in nitrogen, sublimated, freeze-fractured and coated for SEM imaging. SEM confirmed the porous and fibrillar structure of the hydrogels, without evident differences between treatments (Fig. 6). Some areas displayed inhomogeneity in the density of fibres.

### Cytocompatibility

**Figure 7:**
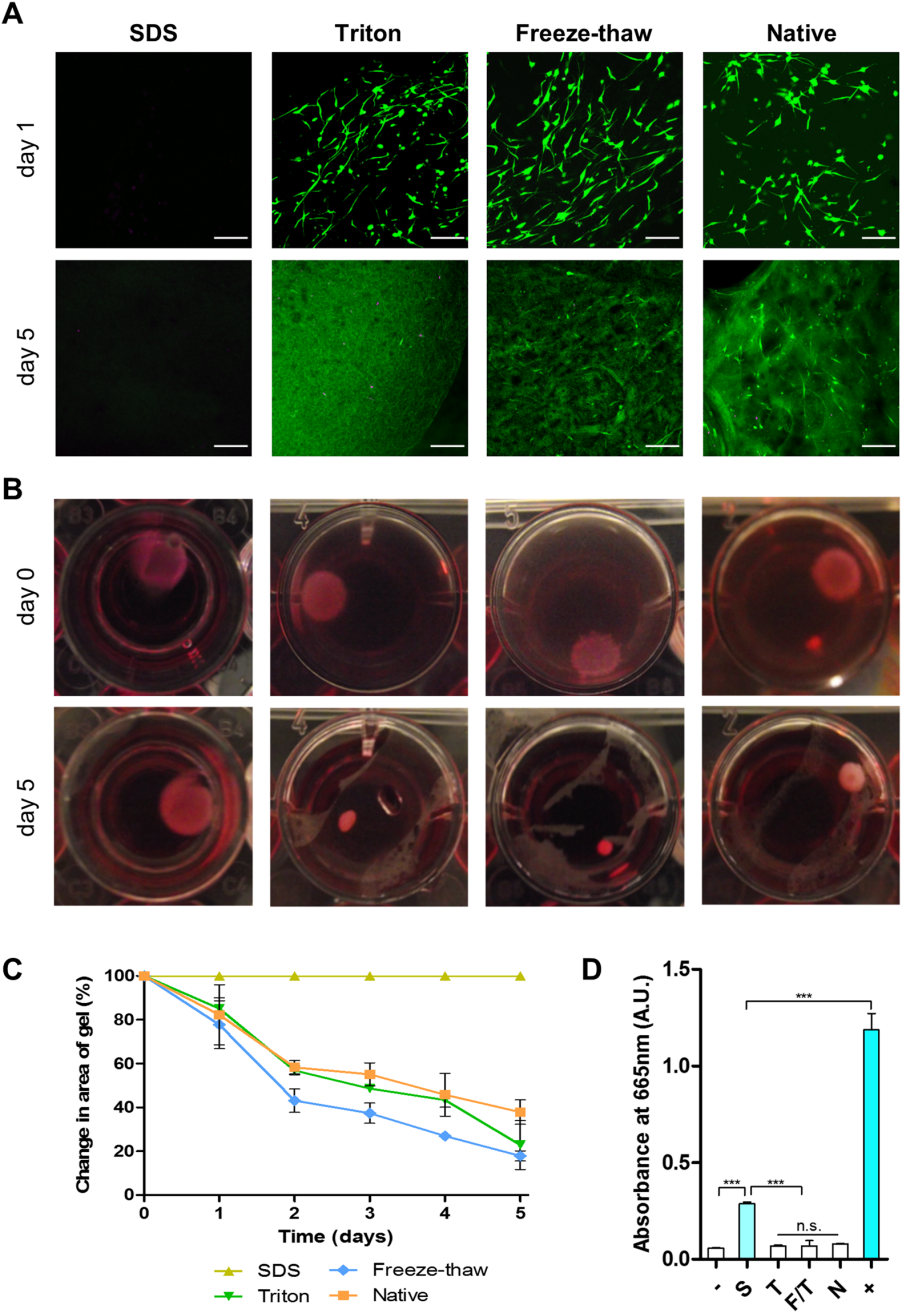
Cell activity in ECM-derived hydrogels: (**A**) Cell viability assessment (green = live, magenta = dead, scale bar = 200 µm); (**B**) macroscopic images of cell-laden hydrogels over time in culture; (**C**) quantification of hydrogel area over time; (**D**) quantification of methylene blue absorbance in the organic phase; *p < 0.05, **p < 0.01, ***p < 0.001.

Human corneal stromal cells were embedded in the hydrogels to examine their cytocompatibility. Cell viability was assessed via calcein-acetoxymethyl ester and ethidium homodimer staining (Fig. 7A). After 1 day in culture, cells were highly viable in the Triton, freeze-thaw and native control hydrogel groups, while no viable cells were visible in the SDS treated group. Healthy cells presented an elongated morphology with small processes, indicating adhesion to the fibrillary architecture of the hydrogels. Over 5 days in culture, the hydrogels underwent significant contraction reflecting the ability of viable cells to actively attach and remodel the hydrogel (Fig. 7B,C). As expected from the viability assessment, the SDS hydrogel group did not undergo contraction. Hydrogels obtained from the SDS decellularization protocol were cytotoxic presumably due to inefficient washing after decellularization. To confirm this hypothesis the presence of detergent residues was determined using a methylene blue active substances (MBAS) assay, which is widely used in water quality control^39^. This assay is based on the binding of the cations in methylene blue with the anions from the detergent that are extracted into the organic phase when in contact with chloroform. Methylene blue extraction confirmed the presence of SDS remnants in the hydrogel thus would explain their cytotoxicity (Fig. 7D).

## Discussion

ECM-derived hydrogels offer great promise as biomaterials for tissue engineering as they can be delivered to the site in need in a minimally invasive manner, allow cell encapsulation prior to delivery, but can also allow for neighbouring cell recruitment. ECM hydrogel solutions have also been used as a bioink for 3D bioprinting several different tissues and organs^29,40,41^. In this study, ECM-derived hydrogels were obtained from corneas decellularized using three different methods. All decellularization methods decreased DNA significantly and retained collagen and other ECM components. All hydrogels were highly transparent, with the freeze-thaw group showing the best optical properties. Gelation kinetics were affected by the decellularization method employed but not the rheological properties. Hydrogels presented a porous and fibrillary structure. Hydrogels were highly cytocompatible when Triton and freeze-thawing methods were used for decellularization, but cytocompatibility was compromised when using SDS as decellularization agent. This work highlights the influence that the decellularization process has on final properties of ECM-derived hydrogels.

One of the main benefits of using ECM-derived hydrogels is the ability to retain multiple ECM components that may not be present in other natural or synthetic hydrogels and therefore more closely mimics the native tissues. Composition analysis of the digested materials via SDS-PAGE confirmed the presence of multiple ECM components when compared to rat tail collagen which just consists of collagen type I. Keratocan, a small leucine-rich proteoglycan almost exclusively found in the cornea, was detected in the ECM-derived hydrogels, irrespective of the decellularization method used. However, this study did show that the choice of decellularization technique is important with SDS decellularization retaining less sGAG than the other techniques tested. This removal of sGAG following decellularization has been previously reported for cornea^14,18,42,43^, cartilage^29,44^, ligament^45^ and adipose tissue^29^. While this study demonstrated that the ECM composition was affected by the decellularization technique used, other techniques may be used to identify more specific tissue or organ ECM components such as mass spectrometry^46–49^ or enzyme-linked immunosorbent assays (ELISA)^32,33,50^.

One potential limitation with using ECM-derived hydrogels is that GAGs and various collagen types, such as collagen type V have been shown to interfere in collagen type I self-assembly *in vitro*^51–53^. *In vivo* small leucine-rich proteoglycans, such as keratocan and decorin in the cornea, play an important role in collagen fibrillogenesis, in terms of collagen assembly nucleation and linear and lateral fibril growth^54^. Therefore, the difference in gelation kinetics between the commercially available collagen type I and the ECM-derived hydrogels can be explained by the presence of ECM components other than collagen type I. Studies from ECM-derived hydrogels from other sources have reported a delay in fibrillogenesis (lag phase) similar to what was shown here. For example, hydrogels obtained from demineralized and decellularized bone showed a short lag phase of around 9 minutes^55^, while myocardium ECM presented a long lag phase of 40 minutes^56^. Hydrogels obtained from urinary bladder matrix^57^, dermis^19^ and pancreas^58^ presented lag periods in a similar range to the ones reported in this study, between 15 and 25 minutes. Furthermore, the presence of detergent remnants might have an influence in the increased gelation time seen in SDS hydrogels. When we attempted to use concentrations above 0.1% SDS for decellularization, it was found that hydrogels could not be formed. This is in agreement with findings from Gaetani and colleagues who could not fabricate pancreas ECM-derived hydrogels when they used 1% SDS for decellularization^58^.

Pre-gel solutions presented shear thinning characteristics, i.e. viscosity decreases as shear rate increases. Values presented here are in accordance to those reported for ECM-derived hydrogels from myocardium^56^, dermis^19^, urinary bladder matrix^57^ skeletal muscle^24^ and cornea^50^. This characteristic offers the potential for these gels to be used as an injectable biomaterial and for their use as bioinks in 3D bioprinting^29–34^. Gelation profiles seen with turbidimetric analysis were also obtained when using rheology. Despite being more concentrated than the rat tail collagen hydrogels, the cornea ECM-derived hydrogels were softer. However, these values are in a similar range to the ones found in hydrogels derived from other tissues^55,57^. The values are lower than those reported for the storage and loss moduli of the native cornea, which are 2 kPa and 0.3 kPa, respectively^59^. Additional steps such as cross-linking^60^ may be required to increase the modulus of the hydrogels to match the native corneas.

In this study, cryoSEM was used to investigate the ultrastructure of the hydrogels. This technique is believed to be better at retaining the hydrogel’s structure compared to conventional SEM as the water present in the highly hydrated hydrogels is sublimated at extremely low temperatures^61^. The hydrogels obtained here were highly fibrillar and porous, which closely resembled the structure reported for ECM-derived hydrogels from other tissues, such as dermis^19^, myocardium^56^, demineralized bone^55^ and small intestinal submucosa^35^. These studies imaged the hydrogels using conventional SEM after glutaraldehyde fixation and critical point drying of the samples. Johnson and colleagues also described the presence of areas of higher fibre matrix density than others, which prevented implementation of automatized pore size quantification^56^.

In the current study, standard gelation parameters where used that can influence the hydrogels properties if modified. Johnson and colleagues studied the effect of temperature, ionic strength, pH and ECM concentration on the fibril architecture, mechanical properties and gelation kinetics of myocardium ECM-derived hydrogels^56^. They showed that no hydrogels could be formed at 4 °C and 22 °C, while at 37 °C they obtained robust hydrogels. Fibre diameter was not influenced by any of the conditions studied. Similar to our results, the authors reported areas of increased fibre density visualized by SEM. The effect that reduction of ionic strength to 0.5x PBS was striking as it increased mechanical properties and sped up gelation. pH did not influence any of the analysed parameters. Increase in ECM concentration increased mechanical properties and viscosity as reported for urinary bladder matrix^57^, bone^55^ and dermis^19^.

Furthermore, tissue origin plays an important role in hydrogel characteristics. It has been shown that porcine myocardium hydrogels retain more sGAG and have increased strength than healthy human myocardium hydrogels^49^. While using human tissues would ease the translation into the clinic as the issue of xenoimmunogenicity is avoided, sourcing healthy organs is difficult as these would be required for transplantation. However, for the cornea specifically, human corneas deemed unsuitable for transplantation due to low endothelial cell count, have the potential to be used to manufacture hydrogels. Decellularized porcine corneas have been used clinically as alternatives to donor grafts^62,63^, paving the path for other treatments based on ECM-derived materials.

Cells embedded within the hydrogels presented high viability and adopted a spindle morphology with multiple processes, indicating good adhesion to the fibres, except in the SDS group. Over the culturing period, the hydrogels contracted, as reported by Wolf and colleagues using dermal ECM-derived hydrogels^19^. This contraction effect was due to the traction forces exerted by the cells on the collagen fibrils. However, when cells died as in the SDS case, the hydrogels retained their shape and size. Depending on the application, the rate of contraction might limit the usefulness of these hydrogels without further cross-linking to strengthen them^60^ or using a cell culture condition that inhibits contractile behaviour^64,65^. Furthermore, contraction can also be inhibited when the hydrogel adheres to a material or is constrained at the edges^66^. When the hydrogel adheres to the tissue matrix, it will be less able to contract compared to a free-floating hydrogel in culture medium.

In this study, all steps were performed under sterile conditions so that no final sterilization method was required. However, for clinical purposes authorities may require terminal sterilization. It has been shown that common sterilization techniques such as electron beam, gamma irradiation or ethylene oxide inhibit gel formation when performed on the powder, but not when the lyophilized digest is treated^12^. Furthermore, sterilizing the lyophilized digest could increase the likelihood of translation into the clinic as a ready-to-use product, whereby the clinician can rehydrate the lyophilize digest with a basic salt balanced solution and let the hydrogel form *in situ*.

Future studies are required to assess the suitability of these hydrogels before they can be used for clinical applications. The hydrogels will need to be tested *in vivo* to evaluate the immunological response and ability to integrate with the surrounding tissue. The survival and functionality of the hydrogels would also need to be monitored. Should the hydrogel not induce any negative effects *in vivo* then the potential for hydrogels to replace damaged or diseased tissue could be explored using suitable animal models. For example in the cornea, the potential for ECM-derived hydrogels to replace tissue damaged due to trauma or diseases such as keratoconus could be explored. This is important due to a global shortage of donor corneas available for transplantation^67^.

In summary, here we demonstrated the importance that the decellularization method has on the final characteristics of ECM-derived hydrogels using corneas as tissue model. Similar phenomena would be expected for hydrogels derived from the ECM of other tissues although the precise decellularization protocol should be specific to the tissue type. We would therefore recommend that researchers or companies involved in the development of ECM-derived hydrogels examine different decellularization techniques to find the optimal approach for preparing their hydrogels.

## Materials and Methods

### Decellularization of porcine corneas

Porcine ocular globes were obtained from a local slaughterhouse. The remaining pieces of flesh were removed and, under aseptic conditions, the eyes were immersed in 2% iodine solution (Videne, Ecolab, Belgium) in sterile phosphate buffer saline (PBS) for one minute, gently rocking throughout. The eyes were subsequently washed twice in sterile PBS and the central corneal button was excised using scissors and cut into small pieces (2 mm × 2 mm, approximately). Three decellularization methods were tested:

- SDS (anionic detergent): each corneal button was immersed in 3 ml of 0.1% (w/v) sodium dodecyl sulphate (SDS, Sigma-Aldrich) solution for 72 hours under rotation. Solution was exchanged every 24 hours.
- Triton (non-ionic detergent): each corneal button was immersed in 3 ml of 1% (v/v) Triton X-100 (Sigma-Aldrich) solution for 72 hours under rotation. Solution was exchanged every 24 hours.
- Freeze-thaw (mechanical procedure): each corneal button was immersed in 5 ml sterile deionized H_2_O and placed in a −80 °C freezer for a minimum of 5 hours. Thereafter, they were let to thaw at room temperature. Once thawed, the solution was exchanged and the procedure repeated until 5 freeze-thaw cycles had been completed.

Afterwards, all corneas where subjected to a DNAse treatment for 1 hour at 37 °C under rotation. DNAse (Sigma-Aldrich) was used at a concentration of 10 U/ml prepared in 10 mM magnesium chloride buffer at pH 7.5. Corneas where extensively washed with sterile deionized water for 72 hours, with solution exchanged every 24 hours, under gentle rotation. Finally, decellularized corneas where dehydrated using a freeze drier and turned into powder by cryomilling (SPEX SamplePrep Freeze/Mill). Non-decellularized corneas were lyophilized and cryomilled to be used as controls (native).

### Hydrogel formation

**Figure 8:**
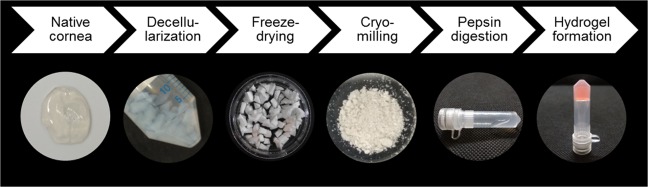
Main steps in the fabrication of cornea ECM-derived hydrogels.

ECM hydrogels were prepared as previously described^68^. Briefly, ECM powder was dissolved in 1 mg/ml pepsin solution in 0.1 M hydrochloric acid at a concentration of 20 mg/ml and incubated for 72 hours at room temperature under slow rotation. Hydrogels were formed by neutralizing the solution with 1 N NaOH, balancing salt concentration using 10x PBS and incubating at 37 °C for one hour to induce fibrillation. Hydrogels had a final ECM concentration of 16 mg/ml. This process is depicted in Fig. 8. Rat tail collagen type 1 hydrogels were fabricated as described previously^68^ and used as a control for some studies.

### Biochemical quantification

Quantification of dsDNA, sGAG and collagen was performed after papain digestion of 100 µl hydrogels. DNA was quantified with the Quant-iT PicoGreen dsDNA Assay Kit (Invitrogen), following manufacturer’s specifications. sGAG quantification was performed using dimethylmethylene blue dye-binding assay (Blyscan, Biocolor). Collagen content was inferred by measuring the content of hydroxyproline using a chloramine T assay^69,70^.

### Histology

Hydrogels were fixed in 4% paraformaldehyde and processed for wax embedding. 6 µm-thick slices of each sample were cut, attached to a glass slide, dewaxed, rehydrated and stained as follows. Haematoxylin & eosin was used to visualize any nuclei still present after decellularization. Slides were stained with Harris Haematoxylin (Sigma-Aldrich) for 4 minutes, followed by a 10-minute wash with running tap water. Then slides were immersed in acid alcohol for 30 seconds and washed with tap water for 5 minutes. Finally, slides were stained with Eosin Y (Sigma-Aldrich) for 2 minutes. Picro-sirius red was used to assess collagen distribution. Slides were stained with Sirius Red (Sigma-Aldrich) in a saturated aqueous solution of picric acid for one hour and then for one minute in 0.5% acetic acid. Alcian blue was used to assess sGAG content. Slides were stained with 1% Alcian Blue 8GX (Sigma-Aldrich) in 0.1 M HCl for 5 minutes followed by three 30-second washes in dH_2_O. After staining, all slides were dehydrated and coverslipped using DPX.

### SDS-PAGE and western blot

7.5% polyacrylamide gels were cast and loaded with 50 µg of sample mixed with Laemmli buffer (1:1) that was previously boiled for 5 minutes. Gels were run for 90 minutes at 120 V. Gels were then stained with GelCode (Thermo-Fisher) following manufacturer’s directions. For western blot, 100 µg were loaded into precast 12% SDS-PAGE gels (Biorad) and run at 200 V for 45 minutes. Gels were transferred to a PVDF membrane using a semi-dry transfer system (Thermo-Fisher) with Pierce 1-step transfer buffer (Thermo-Fisher). The membrane was activated with methanol and then blocked overnight at 4 °C with 3% BSA. Primary antibody (keratocan, sc-66941, Santa Cruz) was used at a dilution of 1:200 in 3% BSA and incubated overnight at 4 °C. Three 5-minute washes with TBST were performed under agitation, and then the HRP-linked secondary antibody (A0545, Sigma-Aldrich) at a dilution of 1:1000 was incubated for an hour at room temperature. Another set of washing steps was carried out and then membranes were developed with Western Chemiluminescent HRP Substrate (Fisher Scientific). Membranes were imaged with GelDoc (Biorad).

### Transparency

The macroscopic appearance and transparency of the gels was assessed by placing them over printed text. Subsequently, light transmittance was quantified. The absorbance of light at several wavelengths ranging from 350 to 700 nm was determined with a microplate reader (BioTek Synergy HTX). Deionized water was used as a baseline control. The transmittance of light was calculated using the following formula:

$$ \% Transmittance={10}^{2-Absorbance}$$

### Gelation kinetics

Gelation kinetics was determined via turbidimetric spectrophotometric analysis, as described elsewhere^57^. Briefly, 100 µl hydrogels were casted into 96-well plates at 4 °C and inserted in a plate reader pre-heated at 37 °C (BioTek Synergy HTX). Absorbance at 405 nm wavelength was measured every 3 minutes for 90 minutes. Absorbance values were normalized with the following formula:

$$NA=(A-{A}_{0})/({A}_{max}-{A}_{0})$$

where *NA* is the normalized absorbance, *A* is the absorbance at any given time, *A*_0_ is the initial absorbance and *A*_*max*_ is the maximal absorbance.

The lag phase (t_lag_) was calculated by obtaining the linear portion of the curve and extrapolating the time value at which the normalized absorbance is 0. Similarly, t_1/2_ was determined as the time at which the normalized absorbance is 0.5. The slope of the linear portion of the curve determined the gelation speed (S).

### Rheology

All rheological experiments were performed using a MCR 102 rheometer (Anton Paar, Austria) equipped with temperature controlling systems and using a 25 mm diameter parallel plate. Viscosity of pre-gel solutions was measured by performing a frequency sweep, from 0.1 to 1000 Hz, at 5% strain at 15 °C. Viscosity constants can be found in Supplementary Table S1. Storage and loss moduli were calculated with fixed frequency of 1 rad/s and 5% strain. Pre-gel solutions were applied at 4 °C and left equilibrate during 10 minutes, after which temperature was raised to 37 °C to induce gelation. Measurements where stopped once G’ values plateaued.

### CryoSEM

Hydrogels were snap-frozen in nitrogen for 5 seconds, sublimated for 40 minutes at −100 °C and 10^−5^ Pa, freeze fractured and sputter coated with platinum for 20 seconds. These were then imaged with a scanning electron microscope at 5 kV (Ultra 2 Zeiss, Germany, with a Quorum Technologies CryoSEM Preparation System, UK).

### Cell culture

Human corneal stromal cells were isolated and cultured as previously described in accordance with the Declaration of Helsinki^64^. The use of human cornea tissue with donor consent for isolating cells received ethical approval from the Trinity College Dublin, University of Dublin, School of Medicine Research Ethics Committee. 100,000 cells were embedded in each 100 µl hydrogel and cast in the wells of a 96-well plate. After gelation, hydrogels were released from the wells and transferred to 24-well plates. Constructs were fed every second day with low glucose DMEM (Hyclone) supplemented with 10% FBS, 100 U/ml Penicillin 100 µg/ml Streptomycin (both Gibco). Cell viability was assessed at day 1 and day 5 by staining the constructs with 2 µM calcein-acetoxymethyl ester and 4 µM ethidium homodimer-1 in PBS for 1 hour at 37 °C in a humidified incubator. Cells were then imaged via laser scanning confocal microscopy (Leica SP8). Furthermore, the shape of the hydrogels was monitored and images were taken daily over 5 days. The area of the hydrogels was calculated using Image J (NIH) and was plotted as the percentage of change of area with time.

### Methylene blue active substances (MBAS) assay

MBAS assay was performed with some modifications from previously described methods^71^. A methylene blue (Sigma-Aldrich) solution was prepared in water to a final concentration of 250 µg/ml. 1 mg of cryomilled powder from each experimental group was mixed with 1 ml of distilled water and vortexed thoroughly for 1 minute and spun down for 30 seconds on a mini-centrifuge. 250 µl of this supernatant was mixed with 250 µl methylene blue solution and vortexed. Then 1 ml of chloroform was added, vortexed 3 times for 30 seconds and centrifuged for 1 minute using a benchtop centrifuge. A negative control was obtained using 250 µl distilled water and a positive control using 250 µl of 0.5% SDS solution. A phase separation was evident in all tests and visually the SDS group had a blue coloration in the organic (bottom) phase. This was further quantified by measuring the absorbance of the bottom phase at 665 nm using a plate reader (BioTek Synergy HTX).

### Statistics analysis

GraphPad Prism Software 5.0 (GraphPad Software, Inc. La Jolla, CA, USA) was used to perform statistical analyses. All data are presented as the mean ± SD. One-way ANOVA with Tuckey post-hoc analyses were performed to determine statistical significance. Differences were considered to be statistically significant at p ≤ 0.05.

## Supplementary information

Supplementary Information
