# Author Correction: The impact of decellularization methods on extracellular matrix derived hydrogels

**DOI:** 10.1038/s41598-019-56283-4

**Published:** 2019-12-19

**Authors:** Julia Fernández-Pérez, Mark Ahearne

**Affiliations:** 1 Department of Mechanical and Manufacturing Engineering, School of Engineering, Trinity College Dublin, the University of Dublin, Dublin, Ireland; 2 Trinity Centre for Biomedical Engineering, Trinity Biomedical Science Institute, Trinity College Dublin, the University of Dublin, Dublin, Ireland

Correction to: *Scientific Reports* 10.1038/s41598-019-49575-2, published online 17 October 2019

This Article contains errors in Reference 46 which was incorrectly given as:

Odorico, J. S. et al. Extracellular matrix scaffold and hydrogel derived from decellularized and delipidized human pancreas. Sci. Rep. 8, 1–16 (2018)

The correct reference is listed below as Reference 1:
