# Supplementary material for: The impact of decellularization methods on extracellular matrix derived hydrogels
Source: Sci Rep. 2019 Oct 17;9:14933. doi: 10.1038/s41598-019-49575-2 (PMC6797749; doi:10.1038/s41598-019-49575-2)
Supplement: Supplementary file 1 — Supplementary Information [file 41598_2019_49575_MOESM1_ESM.docx]

**Supplementary Material**

**The impact of decellularization methods on cornea extracellular matrix-derived hydrogels**

Julia Fernández-Pérez^1,2^ and Mark Ahearne^1,2*^

^1^ Dept of Mechanical and Manufacturing Engineering, School of Engineering, Trinity College Dublin, University of Dublin, Ireland

^2^ Trinity Centre for Bioengineering, Trinity Biomedical Science Institute, Trinity College Dublin, University of Dublin, Ireland

* Corresponding Author:

Mark Ahearne

Tel.: +353 1 8962359

Fax: +353 1 6795554

E-mail address: ahearnm@tcd.ie

**Viscosity measurements**

| Condition | k | n | r^2^ |
| --- | --- | --- | --- |
| SDS | 0.3385 ± 0.09793 | -0.2045 ± 0.02835 | 0.9128 ± 0.07876 |
| Triton | 0.3394 ± 0.05396 | -0.3169 ± 0.07344 | 0.9727 ± 0.0225 |
| Freeze-thaw | 0.5912 ± 0.3086 | -0.3008 ± 0.03451 | 0.9713 ± 0.02434 |
| Native | 0.3385 ± 0.09793 | -0.2839 ± 0.08776 | 0.9178 ± 0.1014 |

The data from the complex viscosity vs frequency was fit to a power-law as follows:

$$\left| \eta^{*} \right|=kf^{-n}$$

where $\eta^{*}$ is the complex viscosity, $f$ is the frequency, and $k$ and $n$ are constants.

Supplementary Table 1. Constants from the complexity viscosity measurements. Average ± SD.
